# Caring for Carers: Positive and Normative Challenges for Future Research on Carer Spillover Effects in Economic Evaluation

**DOI:** 10.1016/j.jval.2018.10.010

**Published:** 2019-05

**Authors:** Padraig Dixon, Jeff Round

**Affiliations:** 1 Population Health Sciences, Bristol Medical School, University of Bristol, Bristol, England, UK; 2 MRC Integrative Epidemiology Unit, University of Bristol, Bristol, England, UK

**Keywords:** carers, economic evaluation, equity, spillover effects

## Abstract

**Background:**

Many individuals rely on family and friends to provide care outside of the formal healthcare sector. The need for caring is driven by many factors, including government policies toward health and social care, and increased prevalence of chronic and comorbid conditions. Informal care may give rise to “spillover” effects from the health of a cared-for individual to the health of carers. Spillover effects are rarely reflected in economic evaluations, in spite of growing research interest in this area, and recommendations from bodies such as the National Institute for Health and Care Excellence (NICE) and the Second Panel on Cost-Effectiveness that effects of this type be included in cost-effectiveness analysis.

**Objective:**

We explore the positive and normative issues to which the inclusion of carer spillover effects in economic evaluation may give rise and how future research might begin to address these challenges.

**Results:**

Positive challenges include the identification of causal rather than coincidental impacts on carers, selection into caring, and the measurement and treatment of spillover effects. The normative issues are related to these challenges, and particularly include impacts on equity, and spillovers that improve rather than reduce the health of carers.

**Conclusions:**

We argue that challenges including spillover effects in economic evaluation are considerable. Avenues for future research and possible solutions to these challenges include a re-orientation of analytic perspectives so that the impacts of caring on carers are accounted for where appropriate, and the design of studies to collect robust evidence to inform debate and guidance development in this area.

## Highlights

• Many individuals rely on informal care from friends and family, which may give rise to “spillover” effects in which the health of the carer is affected by changes in the health of the cared-for individual.
• The challenges of including these spillover effects in economic evaluation are likely to be considerable, and include the identification of causal rather than coincidental impacts and possible impacts on health equity.
• Care needs to be taken that the treatment of spillover effects does not inadvertently reduce patient health.

## Introduction

Changes in the health of an individual may have profound consequences for family, friends, and others. One important and frequently encountered consequence is the need for informal caregiving by family and friends. We define informal caregiving as unpaid support and assistance to an individual living with illnesses or disabilities that may be chronic or acute^1^ and that is above what might normally be expected in the relationship concerned.^2^, ^3^ By contrast, formal care comprises remunerated support provided by professional caregivers working in the health sector.^4^

Many individuals rely on informal caregiving, estimates of the monetary value of which are similar to the value of all formal care.^5^, ^6^, ^7^ The need for caring is driven by a number of factors, including aging populations living with morbidity,^8^, ^9^ changes in family composition,^10^ government policy toward health and social care,^11^ and an increased prevalence of chronic and comorbid conditions.^12^ Informal care may give rise to “spillover” effects on carers arising from the health or circumstances of a cared-for individual or patient. Population-level healthcare decisions should account for the opportunity costs borne by unidentified fellow citizens,^13^ and scrutiny of these effects and their implications for resource allocation is merited.

The possibility of considering spillover effects in cost-effectiveness analyses conducted from a societal perspective was explored in 1996 by the influential “Panel on Cost-Effectiveness in Health and Medicine.”^14^ The panel suggested that sensitivity analysis be undertaken to measure the consequences of spillover effects on caregiver quality of life. The subsequent 2016 Panel report^15^ proposed that such effects, if material, be included in analyses from the societal perspective: “The scope of a study should be defined broadly enough to encompass the full range of groups of people affected by the intervention and all important consequences.”^16^ The National Institute of Health and Care Excellence (NICE) guidelines for the health technology appraisal^17^ recommended the inclusion of “direct health effects, whether for patients or other people” in their reference case for analyses conducted from health system perspectives; nevertheless, no practical guidance was made available to give effect to this recommendation.

Despite increasing methodological and applied work (eg, Al-Janabi et al^18^), spillover effects are infrequently included in economic evaluation,^19^ rare instances of influential examples (eg, Christensen et al^20^) notwithstanding. We build on recent work in this area by exploring the positive and normative issues to which inclusion in economic evaluation of carer spillover effects may give rise and identify areas for future research, the need for which was noted by Neumann et al.^15^

We refer to individuals living with illnesses or disabilities who are the targeted beneficiaries of interventions subject to economic evaluation as *patients*, and those providing informal care (in the sense defined above) to these patients as *carers*. We do not consider health spillover effects owing to mechanisms such as the transmission of infectious diseases arising from vaccination (see Benjamin-Chung et al^21^ for a discussion of some of these issues).

Finally, the spillovers with which we are concerned here relate only to carer health, rather than measures of welfare other than health, such as carer consumption, employment, or leisure. This approach—of focusing on health as the primary object of interest—differs from welfarist analyses of spillover effects, such as that of Basu and Meltzer.^22^ Policy evaluation in which the welfare of individuals is assessed in terms of the self-assessed, subjective, rational, and consistent utilities that these individuals obtain from consumption may be described as welfarist.^23^, ^24^ Cost-benefit analysis is an example of this approach.

The approach of this article is more consistent with extra-welfarist approaches such as cost-utility analysis, which supplement the individual utilities of the welfarist approach with other “nonutility” objects that are relevant to the individual and which should be accounted for in comparing individual outcomes. Interpersonal comparisons of well-being may be made on the basis of these “extra” dimensions of individual well-being.^25^, ^26^

For our purposes, we do not describe what ought to be included when comparing outcomes, but simply claim that including spillover effects does not appear to be in obvious conflict with extra-welfarism. It seems plausible that an extra welfarist decision maker, including under a social decision-making interpretation of extra welfarism,^27^ with objectives other than seeking Paretian welfare improvements,^28^ could consider spillover effects in seeking to optimize some health-related objective function.

## Positive Issues

In this section we describe the conditions necessary for spillovers to be accounted for in a consistent manner. We assume that any analysis proceeds from an analysis of the opportunity costs to which spillover effects may give rise.

### Causal Effects

Analyses of spillovers may be confounded by concurrent but unrelated health changes in the carer, an issue likely to be particularly acute in older, frailer, and comorbid carer populations. Much recent research has relied on cross-sectional study designs that mean it is not possible to establish the temporality of the relationship.

In one recent overview of literature, Tilford and Payakachat^29^ did not identify any studies that included family spillover effects with the framework of prospective analysis of randomized controlled trials (RCTs). Tilford et al^30^ studied autism-related sleep disorders and quality-of-life outcomes for primary caregivers and concluded that “Our results are not intended to suggest a causal relationship between sleep and depression, as we recognize the limitations of cross-sectional data and the potential for relationships to be recursive.” Adjustment using multivariable regression, or similar techniques such as matching, cannot eliminate the possibility of residual confounding or collider bias (discussed further below).

If study designs are not prospective, and particularly if they are not based on randomized controlled trials, it will be difficult to establish if an observed change in carer health is due to a spillover effect, and the observed association may not be causal. This could lead to a type of double counting (or undercounting) in which a combined estimate of health change (patient plus carer via a spillover effect) is estimated with error; for example, a cross-sectional study design that cannot identify temporal changes might estimate spillover effects by counting changes in carer health that were unconnected to caring and are therefore incorrectly characterized as a spillover.

Moreover, measuring carer status at one point in time, such as in the period immediately after a change in caring status, may be misleading. Carers need not passively accept a decrement to health as an unavoidable consequence of a change in caring status. Any impairment of carer health may in some cases be reduced or eliminated relatively quickly in some cases after the receipt of effective treatment directed at the carer, and the point of observation of these effects is therefore a critical issue in study design.

### Selection Bias

Selection bias is likely to be important in understanding spillover effects. This does not refer to the idea of representativeness but instead to the possibility that, in selected populations, a relationship can exist between an outcome (such as carer health) and an exposure (becoming a carer) when the basis of selecting the study population is itself a potential outcome of both caring and carer health and is conditioned on in analysis. This is sometimes known as collider bias.^31^ This could arise from sampling designs that recruited or solicited information only from, for example, a support group for carers who self-report health impacts from caring, which may create an association between caring and carer health when none exist in truth.^32^

Sterne et al^33^ suggest a means of evaluating bias in nonrandomized studies: to view such studies against the ideal, hypothetical, and potentially unethical randomized trial, conducted on the same group of individuals to answer the question of interest. The ideal hypothetical experiment in the present context would be to randomize individuals into different caring statuses, which would ensure (in expectation) that their assets, capacity to care, and other covariates were balanced and the effect of caring on outcomes such as carer health could be assessed without bias. This hypothetical experiment is indeed impractical and unethical but reveals the challenge faced by empirical research in this field, and the types of bias that are likely to arise in observational studies, because carers are known to differ along a variety of baseline dimensions that will influence the apparent but not necessarily the actual association between caring and carer health.^1^, ^4^, ^34^

The challenges of identifying the causal effects of health conditions and handling selection bias are similar whether a patient-only or patient-plus-carer perspective is adopted. Evidence from all manner of study designs is potentially relevant, although robust randomized study designs are likely to be preferred where feasible. Careful analysis is required to detect spillover effects that are relevant to the context under investigation, given the possibilities for mistaking coincidental for causal impacts, the risks of double counting, and the relative lack of evidence on carer health impacts and spillover effects generally.

### Beneficial Impacts on Carer Health

Spillover effects may improve carer health.^1^, ^35^, ^36^, ^37^, ^38^, ^39^, ^40^ This association, if causal, is consistent with altruistic motivations for providing care (eg, Becker^41^) and with findings^40^, ^42^ that the overall level (or perceived level) of caregiving burden may influence the extent of spillover effects. It is not obvious that there are grounds for excluding positive (health improving) spillover effects from consideration in economic evaluations, although the uses to which those data might be put continues to be the subject of debate.

Economic evaluations are premised on analysis of mean effects in the population concerned because it is generally these effects that are relevant to a decision maker. The mean impact of caring on carers may have a material effect on the probability cost-effectiveness when account is taken of heterogeneity in carer responses, notwithstanding the possibility that caring responsibilities have nuanced effects that depend on circumstances and may, on balance, reduce average carer health.^38^ These heterogenous effects also have implications for the design and sensitivity of instruments used to measure carer health and wellbeing, a point we consider in the “Other Issues” section below.

### Identification of Carer Networks

Identification of carer networks is a necessary step in incorporating spillover effects. Gold et al^14^ note in the context of economic evaluations conducted from the societal perspective: “In the extreme, through altruism, entire communities can be affected.”

Ride^43^ discusses a natural network that arises in the context of postnatal depression—that formed between mother and child. Al-Janabi et al^44^ develop a framework for including spillovers in “family networks”, where the term “family” is “used loosely to describe the close network of individuals around the patient.” Canaway et al^45^ describe an outcome measure for use in economic evaluation that captures care benefits to those close to the dying.

The present article is concerned with the slightly narrower circumstance in which the identity of the carer or carers concerned is unambiguous. Nevertheless, at least one further positive challenge remains once all relevant carer health impacts have been defined: the comparison of interventions that have been evaluated using carer networks of differing composition.

For example, an evaluation of an intervention could include only a primary carer, or it could include each and every carer involved with providing care to a particular individual. Discretion to define the “depth” of these carer networks—even when strictly limited to carers (as defined) rather than wider networks of family and friends—will complicate comparisons of these effects in different disease areas or for different interventions for the same disease because the extent of any health impact will depend on the depth of the spillover network analyzed. This may also give rise to equity issues—if attention is focused on the widest possible network of informal caregivers, it is possible that more health impacts will be calculated for some patients and not others, even if the patient-only and primary caregiver effects are identical. These types of normative complications are discussed further in the “Normative Issues” section below.

### Other Issues

The importance to economic evaluation of heterogeneous carer impacts has implications for the design and sensitivity of instruments used to measure carer health.^46^, ^47^ Wittenberg and Prosser^48^ argue that “The combination of positive and negative effects that is inherent to spillover of illness underscores the need for the measurement instrument used to include the correct definition of domains to capture this effect.”

In a sense, these are familiar arguments concerning instrument suitability, although the challenges may be more severe when considering that health impacts on carers may be a fraction of the impact on the patient.^49^ Al-Janabi et al^50^ report the difficulties involved in predicting carer health status from the health of their cared-for patients. Canaway^51^ describes a number of approaches to understanding and measuring carer impacts in the context of end of life care. Bhadhuri et al^52^ find that both the EuroQol, 5-dimension, five level^53^ instrument and Short-Form, Six-dimension^54^ instrument have a reasonable degree of validity for measuring health spillovers.

In this context of measuring impacts, it is important to consider the likely quantitative significance of spillover effects in different circumstances. Funding and implementing robust study designs in this area will be challenging, but as Al-Janabi et al^55^ argue: “There is both a moral and a practical imperative to consider carers in healthcare decisions given the vital role they have in supporting the health system.” A more direct focus on carers as the central object of investigation is likely to be desirable in some cases, but in others justification as to why carer impacts ought not be included may be appropriate.^48^

A further challenge is accounting for changes in caring status. For example, a carer may change their place of residence, decide to stop caring, require care themselves, or die.^56^ If a prospective evaluation of an intervention determined it to be cost-effective when accounting for spillover effects, should the conclusion of this evaluation change in light of changes in carer status? This is both a positive and a normative challenge. The positive challenge is the need to measure and reflect these changes in an economic evaluation when the data concerned are likely to reflect an observational rather than an interventional study design. The normative issue is of a type explored in more detail in “Normative Issues” section below: if caring status is more likely to change for one group of carers than another, should such differences between compared groups be treated in the same way?

Finally, a fundamental tenet of economic evaluation is that meaningful conclusions depend on interventions being compared to the best-available comparator technology.^57^ The inclusion of spillovers in economic evaluation presents a subtle complication to the selection of relevant comparators, which may require the evaluation of a technology other than the one targeted at the patient who would otherwise be the focus of the cost-effectiveness analysis. What is the best available comparator technology when effects on both patient and carer health are included? If spillover effects are incidental impacts on third parties, is there a case for considering comparators other than the one most appropriate to the patient, even if to do so may reduce the aggregate of patient and carer health?

## Normative Issues

None of the discussion of positive effects can, on its own terms, inform a decision as to whether spillover effects ought to be routinely included in economic analysis, given the normative issues to which including spillover effects in economic evaluation may give rise. In this section, we focus on two normative issues that seem particularly consequential. First, we consider the impact on equity in relation to resource allocation decisions. Second, we consider maximizing population health in the presence of spillovers.

We consider the implications of each issue against principles sometimes used (eg, Cookson and Dolan^58^ and Persad et al^59^) to characterize the justice of healthcare resource allocations: egalitarianism (equal treatment), benefit to the worst-off (Rawlsian-type approaches), need (allocation according to individual need), and maximization (maximizing aggregate population health). In doing so, we do not comment on the desirability of each principle of justice per se, nor do we expect that any system of allocation would perfectly accord with accepted interpretations of these principles, just as existing systems of healthcare resource allocation do not. The exercise is intended to characterize normative issues that merit scrutiny in this context.

### Equity

Accounting for spillover in economic evaluation may challenge equity because care may well be directed at patients with carer and family networks and away from those without. Basu and Meltzer^22^ ask: “Do we want to value the health of married people more than that of unmarried people? What about people who have few or many children or few or many friends?”

This is an example of what Culyer and Bombard^60^ describe “red flags” for considerations of equity in the context of technology assessment, being a behavior and circumstances that “could, at least in principle, disadvantage some people relative to others.” Specifically, accounting for carers may amount to treating *citizen* quality adjusted life years (QALYs) equally, but not *patient* QALYs equally. By citizens we mean everyone in society; the inclusion of spillovers in economic evaluation would therefore represent a departure from the *patient*-centered health system that has otherwise been the foundation of healthcare in countries such as the United Kingdom. This would generally not be consistent—when viewed from the patient’s perspective—with egalitarianism, allocation according to need, and the welfare of the worst-off individual. It may be consistent with maximizing overall societal QALYs of all citizens, a point we return to in the next section.

As Basu and Meltzer^22^ and Al-Janabi^49^ note, not including such claims is itself a decision that may conflict with patient concerns that are neglected in conventional cost-effectiveness analyses. This objection could be characterized as an instance of the trade-off between maximizing aggregate health and delivering acceptable health equity.^61^ The consequences for equity are real but require empirical evidence as to their magnitude and channels of operation. For example, when spillovers are included then, for example, shallower networks of available carers among the old (eg, Schnittker^62^) could give rise to a transfer of resources to the young. The complications of selection into caring, such as financial capacity to enter full-time caring, also have a bearing on these arguments.

Including spillovers may therefore have an adverse impact on equity, particularly for those with limited access to informal care. Nevertheless, including spillovers may support maximization of QALYs across the population, even if this is not consistent with maximizing *patient* QALYs. We consider maximization issues in the next section.

### Spillovers, Heterogeneity, and a Maximizing Approach to Justice

Maximizing aggregate health in the presence of spillovers may reduce the health of patients. Consider the following example. An elderly individual, receiving informal care, develops a life-threatening health condition. An intervention is available that will extend this individual’s life but with the consequence of severe disability and lifetime care requirements. The new postintervention health state then permanently reduces carer self-assessed health because of spillover effects that cannot be ameliorated.

Accounting for both increases and decreases in carer health is required by spillover logic. It is conceivable that a crude “patient plus carer” QALY maximization decision may be to refuse to provide care to this patient, which would be the case if the intervention were only just cost-effective when the patient’s circumstances alone were considered, but not cost-effective when the carer’s health was additionally considered. Is the extra-welfarist decision-maker justified in denying care to the patient? If the answer to this question is no, accounting for spillover effects is not consistent with a health maximizing approach to resource allocation.

This reflects a familiar objection (Posner’s^63^ “moral monstrousness”) to *act* utilitarianism, in that no action and no infringement of individual liberty is impermissible provided that the aggregate utility, happiness, or (in this example) health is maximized as a consequence of that action. This issue arises in several ways once spillover effects are admitted into economic evaluation. Parents transfer more resources to their adult children than vice versa.^5^, ^64^ The resentment by carers of frail elderly patients is associated with carer anxiety and depression.^65^ Nevertheless, it is problematic to deny an effective treatment to a patient simply because so doing might improve the health of the carer.

In the example above, cost-effectiveness fails because of the impact on effectiveness, in which the aggregate of carer and patient health is less than patient health alone. This is not to deny the possibility that a patient without an informal carer may require expensive formal care, and this formal care may itself not be cost-effective because of the impact on costs, as opposed to effectiveness in the previous example. For example, Neumann et al^66^ estimated the cost-effectiveness of an Alzheimer’s disease drug to be more favorable when informal caregiving costs were included because informal caregiving avoided expensive nursing home costs. Both possibilities require recognition and appropriate handling in cost-effectiveness analyses.

Consider Table 1, in which an alternative course of action raises rather than reduces carer health. Scenario A describes an intervention that is not cost-effective (at a particular cost-effectiveness threshold) when only patient outcomes are considered, but which is cost-effective when spillover carer QALYs are included. We ignore uncertainty in this simple example.

**Table 1:** Choice of the appropriate comparator in accounting for spillovers

	Perspective	
	Patient-only perspective	Patient and carer “spillover” perspective
Scenario A—a single intervention compared to usual care		
Incremental cost of the intervention	£2100	£2100
Incremental patient QALYs	0.1	0.1
Incremental carer QALYs	—	0.01
Total incremental QALYs	0.1	0.11
Cost-effectiveness threshold	£20 000 per QALY	£20 000 per QALY
Incremental cost-effectiveness ratio	£21 000	£19 091
Cost-effective?	No	Yes
Scenario B—separate patient and carer interventions compared to usual care		
Incremental cost of patient intervention	£1900	£1900
Incremental cost of carer intervention	—	£300
Total incremental cost of the interventions	£1900	£2200
Incremental patient QALYs	0.09	0.09
Incremental carer QALYs	—	0.03
Total incremental QALYs	0.09	0.12
Cost-effectiveness threshold	£20 000 per QALY	£20 000 per QALY
Incremental cost-effectiveness ratio	£21 111	£18 333
Cost-effective?	No	Yes

QALYs, quality-adjusted life years.

In scenario B, the same patient-carer dyad is involved, but two separate interventions (one directed at the patient, and one at the carer) are considered. In each scenario, the patient-only perspective is not cost-effective. In scenario B, aggregate costs and QALYs are higher, but the two interventions when considered together are nevertheless more cost-effective than the original intervention targeted at the patient. Nevertheless, in scenario B, the patient is worse off in QALY terms than in scenario A, yet aggregate QALYs are higher. Resources are used more efficiently because the incremental cost-effectiveness ratio in the combined patient/carer perspective is lower than that in scenario A. Again, a QALY-maximizing decision maker would be inclined to accept a reduction in patient health, but this may conflict with priority to the worst-off.

The artificial parameters of this example were selected to illustrate the point, and empirical evidence is required. The controlling mind of an extra-welfarist decision maker must account for these types of effect in maximizing population health, a consequence of which is that the *patient* may be worse off under consideration of spillover effects than without, even if dyadic patient-carer health is higher, and even if aggregate societal health is higher.

## Conclusions

Spillover effects occupy a unique but uneasy position in economic evaluation. They feature in one form or another in important guidelines and reference cases but are infrequently included in economic evaluations. An important task in future research will be to quantify the equity impacts of spillovers, particularly because equity in general is arguably an underdeveloped element in economic evaluation and technology appraisal.^67^ The acceptability of equity impacts could be approached and characterized using analytic approaches, including methods recently described in, for example, Asaria et al^68^ and Cookson et al.^69^ Further high-quality evidence from robust study designs is also needed. There seems to be clear need for further guidance in this area, not only because of the methodological challenges issues noted, but also because the salience of spillover effects is likely to increase in the context of aging and comorbid populations, changes in family composition, and new healthcare technologies.
